# Integrative Omics Analysis Reveals the Importance and Scope of Translational Repression in microRNA-mediated Regulation*[Fn FN2]

**DOI:** 10.1074/mcp.M112.025783

**Published:** 2013-04-02

**Authors:** Qi Liu, Patrick J. Halvey, Yu Shyr, Robbert J. C. Slebos, Daniel C. Liebler, Bing Zhang

**Affiliations:** From the ‡Department of Biomedical Informatics, Vanderbilt University School of Medicine, Nashville, Tennessee 37232;; §Jim Ayers Institute for Precancer Detection and Diagnosis, Vanderbilt University School of Medicine, Nashville, Tennessee 37232;; ¶Department of Biochemistry, Vanderbilt University School of Medicine, Nashville, Tennessee 37232;; ‖Vanderbilt-Ingram Cancer Center, Vanderbilt University School of Medicine, Nashville, Tennessee 37232;; **Department of Biostatistics, Vanderbilt University School of Medicine, Nashville, Tennessee 37232;; ‡‡Department of Cancer Biology, Vanderbilt University School of Medicine, Nashville, Tennessee 37232

## Abstract

MicroRNAs (miRNAs) are key post-transcriptional regulators that inhibit gene expression by promoting mRNA decay and/or suppressing translation. However, the relative contributions of these two mechanisms to gene repression remain controversial. Early studies favor a translational repression-centric scenario, whereas recent large-scale studies suggest a dominant role of mRNA decay in miRNA regulation. Here we generated proteomics data for nine colorectal cancer cell lines and integrated them with matched miRNA and mRNA expression data to infer and characterize miRNA-mediated regulation. Consistent with previous reports, we found that 8mer site, site positioning within 3′UTR, local AU-rich context, and additional 3′ pairing could all help boost miRNA-mediated mRNA decay. However, these sequence features were generally not correlated with increased translational repression, except for local AU-rich context. Thus the contribution of translational repression might be underestimated in recent studies in which the analyses were based primarily on the response of genes with canonical 7–8 mer sites in 3′UTRs. Indeed, we found that translational repression was involved in more than half, and played a major role in one-third of all predicted miRNA-target interactions. It was even the predominant contributor to miR-138 mediated regulation, which was further supported by the observation that differential expression of miR-138 in two genetically matched cell lines corresponded to altered protein but not mRNA abundance of most target genes. In addition, our study also provided interesting insights into colon cancer biology such as the possible contributions of miR-138 and miR-141/miR-200c in inducing specific phenotypes of SW480 and RKO cell lines, respectively.

MicroRNAs (miRNAs) are small noncoding RNAs that pair to the messenger RNAs (mRNAs) of protein-coding genes to repress their expression by promoting mRNA decay and/or inhibiting translation (1). In response to miRNA transfection or knockdown, widespread changes in both mRNA and protein abundance have been observed using DNA microarrays and global proteome profiling by stable isotope labeling with amino acids in cell culture, respectively (2–4). Nonetheless, the relative contribution of these two mechanisms to gene repression remains controversial (5). Early studies favor a translational repression-centric scenario (6, 7), whereas recent large-scale studies suggest a dominant role of mRNA decay in miRNA-mediated regulation (2, 3, 8, 9). Because these studies only focused on a small number of miRNAs, mostly through miRNA transfection, a key unanswered question is whether these observations can be generalized to all miRNAs in their endogenous context. Moreover, some studies reached the conclusion based primarily on the response of genes with canonical 7–8mer sites in 3′UTRs (2, 8), and 7–8mer sites have already been reported to enhance mRNA decay (10, 11). Therefore, the conclusion might be biased to the subset of genes with 7–8mer sites and the relative contribution of mRNA decay might be overestimated. A related but more general question is whether sequence features known to drive mRNA decay such as 7–8mer sites are applicable to translational repression.

In contrast to perturbing individual miRNAs, global profiling of miRNA and mRNA expression in multiple systems (*e.g.* tissue types or cell lines) provides a broader approach to investigate the functional relationship between miRNAs and mRNAs. Inverse correlation between miRNA and mRNA expression has been widely used to infer targets susceptible to miRNA mediated mRNA decay (12–16). The key advantage of this approach is that all miRNAs are evaluated simultaneously in their natural biological context. Further integration of matched protein expression data can shed light on the scope of miRNA mediated translational repression; however, proteomic data sets are not readily available for such correlation-based studies.

Here we generated a protein expression data set for nine colorectal cancer (CRC)^1^ cell lines and integrated this data set with matched miRNA and mRNA expression data to perform a comprehensive analysis of miRNA-mediated regulation. We dissected the respective contributions of mRNA decay and translational repression and evaluated four previously reported sequence features for their effect on each type of regulation. We also inferred and categorized miRNA-target interactions, which revealed the relative importance and scope of mRNA decay and translational repression in miRNA regulation and suggested potential roles and downstream effectors of miRNAs in human colon cancer.

## MATERIALS AND METHODS

### 

#### 

##### Cell Lines and Cell Culture

All cell lines were obtained from the American Type Culture Collection (ATCC, Manassas, VA) and grown and harvested within 6 months of date of purchase, or grown from frozen stocks that had been made within 6 months of original purchase. All cell lines were grown in 10% fetal bovine serum and penicillin/streptomycin supplemented medium at 37 °C, 5% CO_2_. DLD-1, HCT-15, COLO 205 and SW480 were grown in RPMI 1640 medium, HCT-116, HT-29 and RKO were grown in McCoy's5A medium, Caco-2 (20% fetal bovine serum) was grown in Minimum Essential Medium and LoVo was grown in F-12 K medium. Cells were passaged 2–3 times per week and harvested at ∼80% confluence. Growth medium was aspirated; cells were washed once in 1 × PBS and collected in 1 × PBS. Cells were centrifuged at 300 × *g* for 5 min and supernatant was discarded. Cell pellets were stored at −80 °C until cell lysis was performed. Three biological replicate cultures were harvested ∼1 week apart from the identical cell culture. For SW480 and SW620 comparison, cells were grown in RPMI 1640 medium. Three biological replicate cultures were harvested ∼1 week apart and these replicates were processed separately and independently through the complete analysis procedure.

##### Cell Lysis, Protein Digestion and Isoelectric Focusing of Peptides

Lysis of cell pellets was performed at ambient temperature. Each biological replicate (one cell pellet from one cell line) was processed in parallel to minimize the effects of systematic errors. Pellets were resuspended in 100 μl of 100 mm ammonium bicarbonate (AmBic) and 100 μl trifluoroethanol was added followed by sonication (3 × 20 s). Samples were incubated at 60 °C and resonicated (3 × 20 s). Protein concentration was estimated using a bicinchoninic acid assay (Pierce, Rockford, IL). Proteins were reduced and alkylated with 40 mm tris(2-carboxyethyl)phosphine /100 mm dithiothreitol and 50 mm iodoacetamide, respectively. Samples were diluted in 50 mm AmBic, pH 8.0 and trypsinized overnight at 37 °C (1:50, w/w). Subsequently, peptides were lyophilized overnight. Peptides were desalted and separated by isoelectric focusing using immobiline IPG strips (24 cm, pH 3.5–4.5) (GE Healthcare) as described (17).

##### Liquid Chromatography-tandem MS

Liquid chromatography-tandem MS (LC-MS/MS) shotgun proteomic analyses were performed on a LTQ XL mass spectrometer (9 cell line panel) or a LTQ velos mass spectrometer (SW480/SW620 comparison) (Thermo Fisher Scientific) equipped with an Eksigent NanoLC AS1 autosampler and Eksigent NanoLC 1D Plus pump, Nanospray source, and Xcalibur 2.0 SR2 instrument control. Peptides were separated on a packed capillary tip (Polymicro Technologies, 100 mm × 11 cm) with Jupiter C18 resin (5 mm, 300 Å, Phenomenex) using an in-line solid-phase extraction column (100 mm × 6 cm) packed with the same C18 resin using a frit generated with liquid silicate Kasil 1. Mobile phase A consisted of 0.1% formic acid and mobile phase B consisted of 0.1% formic acid in 90% acetonitrile. A 90-min gradient was carried out with a 30-min washing period (100% A) to allow for solid-phase extraction and removal of any residual salts. Following the washing period, the gradient was increased to 25% B by 35 min, followed by an increase to 90% B by 50 min and held for 9 min before returning 95% A. MS-MS spectra of the peptides are acquired using data-dependent scanning in which one full MS spectrum (mass range 400–2000 *m/z*) is followed by five MS-MS spectra. MS-MS spectra are recorded using dynamic exclusion of previously analyzed precursors for 60 s with a repeat of 1 and a repeat duration of 1. MS/MS spectra were generated by collision-induced dissociation of the peptide ions at normalized collision energy of 35% to generate a series of b- and y-ions as major fragments.

##### LC-MS/MS Data Analysis and Peptide and Protein Identification

MS/MS scans were transcoded to mzData or mzML file format by Scansifter, an in-house-developed software, which reads MS/MS stored as centroided peak lists from Thermo RAW files. If 90% of the intensity of a tandem mass spectrum appeared at a lower *m/z* than the precursor ion, a single precursor charge was assumed; otherwise, the spectrum was processed under both double and triple precursor charge assumptions. The resulting mzData or mzML files were searched against the Human IPI database (v3.37 with 69,164 entries for the nine CRC cell line set and v3.64 with 84,032 entries for SW480 and SW620 comparison) augmented with potential contaminant protein sequences (keratins, trypsin, IgGs etc.) and with all sequences in both forward and reverse orientation. Searches used the Myrimatch algorithm (version 1.2.11) (18). The database search was configured to consider both fully tryptic and semitryptic peptide matches with a precursor mass/charge (*m/z*) tolerance of 1.25 and a fragment *m*/*z* tolerance of 0.5. Carboxamidomethylation of cysteines was included as static modification and oxidation of methionine as a dynamic modification in the searches.

The IDpicker algorithm (version 2.2.2) (19, 20) was used to assign protein identifications to the set of peptides identified by Myrimatch. For IDPicker analyses, all of the pepXML files generated by Myrimatch for an experiment were combined. This combination of all data within an experiment is essential for accurate estimation of peptide and protein false discovery rate (FDR) and for spectral count-based comparisons of data sets (see below). IDPicker filtered the peptide identifications for each LC-MS/MS run (pepXML file) to include the largest set for which a 5% peptide identification FDR could be maintained. IDPicker allows the user to specify an FDR threshold and then adjusts score threshold accordingly. Peptide filtering employed reversed sequence database match information to determine Myrimatch score thresholds that yielded an estimated 5% peptide identification FDR for the identifications of each charge state, as calculated by the formula FDR = (2 × reverse)/(forward + reverse) (21). For these studies, a 5% peptide FDR was employed.

IDPicker employs a bipartite graph analysis and efficient graph algorithms to identify protein clusters with shared peptides and to derive the minimal list of proteins (19, 20). A bipartite graph analysis technique and parsimony rules were applied to generate a minimal list of proteins that explained all of the peptides that passed our entry criteria. Proteins were required to have at least two distinct peptide sequences observed in the analyses. Indistinguishable proteins were grouped. IDPicker estimates FDR at the peptide-to-spectrum match level and the criteria of 5% peptide-to-spectrum match-level FDR and two peptides per protein are typically used for protein identification. When the sample size in a data set is large, the resulting protein identifications may contain a large number of decoys and thus a high protein-level FDR. In this study, by further requiring a protein identification to be supported by at least 10 MS/MS spectra across the data set, a protein level FDR of 5% was maintained. The IDPicker output with complete peptide identification and protein inference information is available in supplemental Data Set S1.

##### Protein Quantification

Spectral count, or the total number of MS/MS spectra taken on peptides from a given protein in a given LC/LC-MS/MS analysis, was used for protein quantification. Spectral count is linearly correlated with the protein abundance over a large dynamic range. This simple but practical quantification method has found broad application in detecting differential or correlated protein expression (22–26). Here, spectral counts for each protein were normalized by the total spectral count to reduce the variance observed between samples. For total spectral count normalization, the sample with the highest number of total spectral count was chosen and the remaining samples were normalized to it. The normalized data were then log transformed to achieve a better approximation of normal distribution. When multiple proteins are mapped to the same gene, the protein with the largest interquartile range was selected to represent the gene because of its relatively higher expression level and higher variance across the experimental conditions. Protein abundance for each gene in each cell line is available in supplemental Data Set S2.

##### Integrated Proteomic and Transcriptomic Analysis

Matched miRNA and mRNA expression data from the same nine CRC cell lines were downloaded from the Gene Expression Omnibus (GEO, GSE10833, and GSE10843). All cell lines used in these and proteomics studies were obtained from the American Type Culture Collection (ATCC) and maintained in the recommended growth media, allowing a meaningful integration of these data sets. Replicate measurements for protein, mRNA and miRNA expression were highly reproducible (supplemental Figs. S1, S2, and S3). To facilitate data integration, we averaged the abundance from replicates in each cell line for mRNA, miRNA and protein expression data, respectively. For miRNAs, we filtered out those with small expression variance (< = 1) across the nine CRC cell lines and only the remaining 79 miRNAs were included in the subsequent analyses. The proteomics data set covered 5467 genes and the mRNA expression data covered 19,648 genes. We only included the 5144 genes with paired mRNA and protein expression data for the integrative analysis. A significant positive correlation between mRNA and protein abundance was observed for each cell line (supplemental Fig. S4, Pearson's correlation coefficients of log-transformed abundances fell in the range between 0.47 and 0.53, *p* < 2e-16). These correlation coefficients were larger than or similar to previously reported protein-mRNA correlations in human samples (27, 28).

**Fig. 1.**
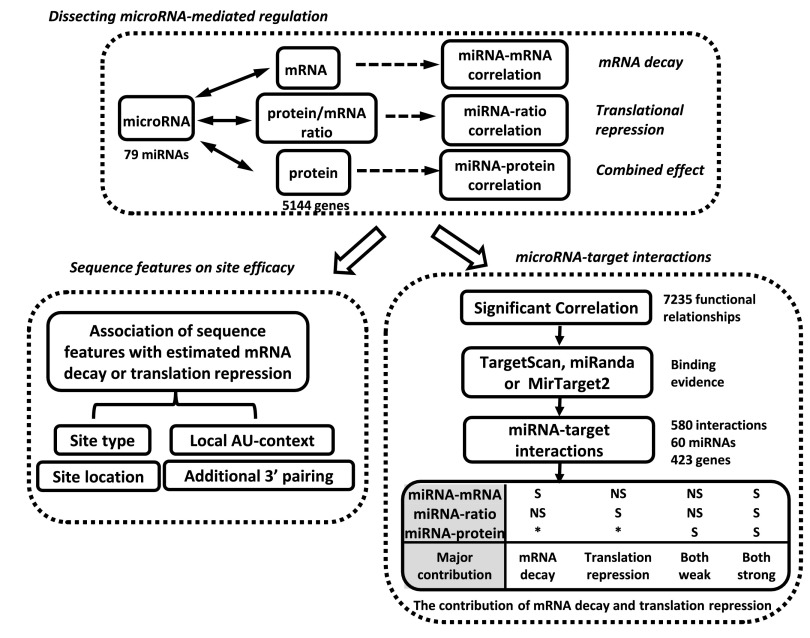
**Overview of the integrative omics analysis.** We calculated three types of correlations between miRNA and genes: miRNA-mRNA, miRNA-protein, and miRNA-ratio. Using the strength of miRNA-mRNA and miRNA-ratio correlations, we estimated the effect of four features on site efficacy in mRNA decay and translational repression, respectively. Meanwhile, we combined functional evidence from the three types of significant inverse correlations (miRNA-mRNA, miRNA-protein, or miRNA-ratio) and binding evidence from sequence-based prediction tools to identify miRNA-target interactions. Finally we classified these interactions into different categories based on the type of supporting correlation and inferred the major contributor (mRNA decay or translational repression) to miRNA-mediated regulation in each category (S: significant; NS: nonsignificant; *: S or NS).

We calculated three types of expression correlations between the 79 miRNAs and the 5144 genes, miRNA-mRNA correlation, miRNA-protein correlation and miRNA-ratio (protein-to-mRNA ratio) correlation (Fig. 1). miRNA-mRNA correlation has been widely used to predict targets susceptible to miRNA mediated mRNA decay (12–16). Protein-to-mRNA ratio is mainly determined by translation efficiency and protein degradation (28). Thus, miRNA-ratio correlation can be used to identify targets susceptible to miRNA mediated translational repression. (We also tried an alternative approach, partial correlation between miRNA and protein expression, which factors out the effect of variation in mRNA abundance, and obtained similar results). Because the impact of miRNA on protein output is a combined result of mRNA decay and translational repression, miRNA-protein correlation can help identify not only targets susceptible to a strong effect of either type of regulation, but also targets affected by modest mRNA decay and translational repression simultaneously. Integrative analysis of these three correlation types allows an estimation of the relative contributions of mRNA decay and translational repression to each miRNA-mediated repression. To test the usefulness of these three correlation types for discovering functional relationships between miRNAs and genes, for each type of correlation, we evaluated the functional coherence of genes correlated with the same miRNA and evolutionary conservation of the 6mer seed match regions of genes correlated with their cognate miRNAs. Results from these analyses demonstrated that a significant inverse correlation (Pearson correlation coefficient<-0.8, *p* < 0.005) of any of the three types helped identify genes that are more likely to be functional miRNA targets (supplemental Text S1, supplemental Figs. S5 and S6). We also performed evaluation based on Spearman's correlation and similar results were obtained.

Using the strength of miRNA-mRNA and miRNA-ratio correlations, we evaluated the effect of four previously reported sequence features on site efficacy in mRNA decay and translational repression, respectively, including type of target site, site location, local AU-context and additional 3′ pairing (Fig. 1). Meanwhile, to infer miRNA-target interactions, we identified miRNA-target pairs supported by both statistically significant correlation (Pearson's correlation coefficient<-0.8, *p* < 0.005) and sequence-based target prediction using TargetScan (29, 30), miRanda (31), or MirTarget2 (32) (Fig. 1). Finally, we categorized the identified interactions based on the type of supporting correlations to infer the relative contributions of mRNA decay and translational repression in miRNA-mediated regulation.

##### Sequence Features

Sequences were downloaded from Ensembl database (Homo sapiens genes GrCh37.p3 data set). R scripts were generated to retrieve 3′UTR, 5′UTR and ORF sequences for all 5144 genes using the biomaRt package (33, 34). When multiple transcripts mapped to a single HGNC gene symbol, only the longest 3′UTR, 5′ UTR and ORF were included in the analysis.

We used the scheme described by Grimson *et al.* (10) for scoring AU-content and additional 3′ pairing. For AU-content, we considered the composition of residues 30nt upstream and 30nt downstream of a seed site, with weighting inversely proportional to the distance from the seed site. For additional 3′ paring, we credited one point for each contiguous pair within the 4mer corresponding to nucleotides 13–16 and one-half point for contiguous pairing elsewhere (10). The position of miRNA 4mer and its complement in the message were allowed to be offset, but a one-half point penalty was assessed for each nucleotide of offset beyond 2nt.

## RESULTS

### 

#### 

##### Proteomics Data for Nine CRC Cell Lines

**Fig. 2.**
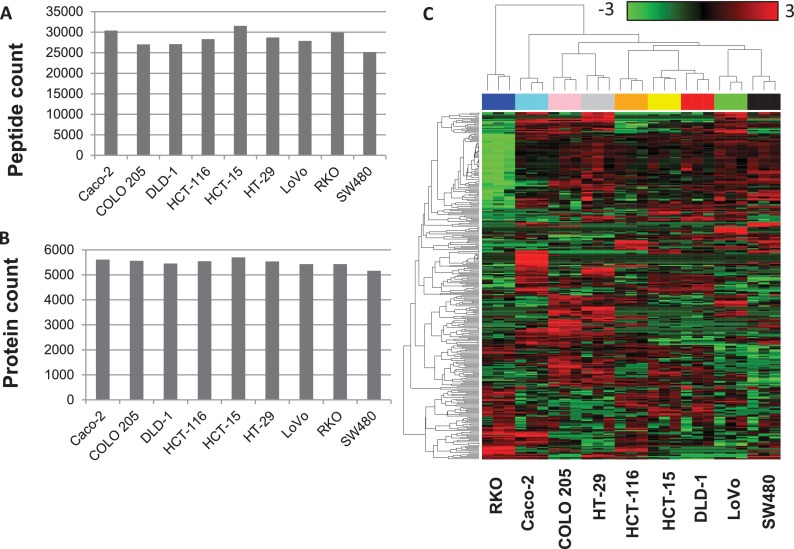
**Shotgun proteomics provided robust global profiles of cellular proteomes.** *A*, Number of peptides identified for each cell line. *B*, Number of proteins identified for each cell line. *C*, Unsupervised clustering indicated high reproducibility of the profiles. The heat map was created based on the top 5% of proteins with the highest variation across all 27 experiments. Each row represents a protein and each column represents an experiment. Samples are color-coded on the top by cell line and labeled at the bottom. The color scale bar shows the relative protein expression level (0 is the average expression level of a given protein in all samples).

We performed three replicate LC-MS/MS-based shotgun proteomic analyses on nine CRC cell lines (Caco-2, COLO 205, DLD-1, HCT-15, HCT-116, HT-29, LoVo, RKO, and SW480). The data set consisted of a total of 6124 proteins with a protein FDR of 5%. The number of unique peptides and the minimum number of proteins that were required to explain the observed peptides in each cell line are shown in Figs. 2*A* and 2*B*, respectively. Although the numbers of peptides identified for each cell line varied from 25,159 to 31,526, the numbers of proteins were very consistent (from 5162 to 5698), suggesting that we were close to the saturation of protein identification in the CRC cell lines within the detection limit of our platform. The Pearson's correlation coefficients between spectral counts of biological replicates fell in the range between 0.9 and 0.98, suggesting high reproducibility and reliability of the protein expression measurements (supplemental Fig. S1). An unsupervised clustering analysis based on the top 5% proteins (*i.e.* 306 proteins) with the largest variation across all 27 experiments provided further evidence for high reproducibility between replicates (Fig. 2*C*). More importantly, the clustering result also revealed distinct proteome profiles for individual cell lines. The tight clustering of HCT-15 and DLD-1, two cell lines isolated from different sites of the same patient, demonstrated that the label-free shotgun proteomics analysis was both robust and sensitive.

##### Sequence Features Affecting Site Efficacy

Previous studies have uncovered a number of sequence features that boost site efficacy, including the type of target site, site positioning within the gene structure, local AU-rich context and additional 3′ pairing. Because changes in mRNA expression after the transfection of miRNA were used to assess site efficacy (10, 11), these identified features could be interpreted mainly to govern mRNA decay. To our knowledge, there is no research reporting the impact of sequence features on site efficacy in translational repression. Here we used the strength of miRNA-mRNA correlation and miRNA-ratio correlation to estimate how these sequence features affect site efficacy in mRNA decay and translational repression, respectively. The 3′UTR length of genes, number of target sites, type of target sites, and scores for local AU-context and additional 3′ pairing for all miRNA-gene pairs were listed in supplemental Data Set S3. Although our correlation-based analysis was not as direct as previous studies using changes in mRNA abundance after the perturbation of miRNA expression, our results on sequence features boosting site efficacy in mRNA decay were consistent with previous reports. In addition, we found that most of these features cannot enhance site efficacy in translational repression except for local AU-rich context.

##### Type of Target Site

**Fig. 3.**
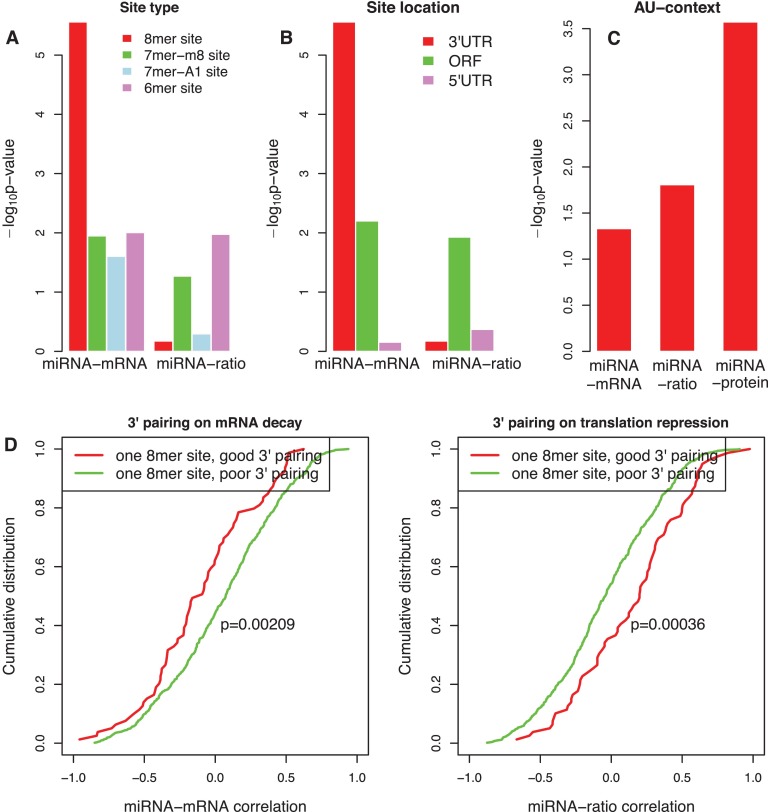
**Sequence features affecting the efficacy of mRNA decay and translational repression.** *A*, Efficacy of different target site types. Plotted are *p* values calculated by one-sided KS-test for comparing the cumulative distribution of miRNA-mRNA and miRNA-ratio correlation between miRNAs and genes with different target site types with those between miRNAs and genes with no site. *B*, Efficacy of sites located in 3′UTR, ORF or 5′UTR. Plotted are *p* values calculated by one-sided KS-test for comparing the cumulative distribution of miRNA-mRNA and miRNA-ratio correlation between miRNAs and genes with 8mer sites located in different gene regions with those between miRNAs and genes with no site. *C*, Efficacy of local AU-content. Plotted are *p* values calculated by one-sided KS-test for comparing the cumulative distribution of miRNA-mRNA, miRNA-ratio, and miRNA-protein correlation between miRNAs and genes with a high AU-content site (top quartile) with those between miRNAs and genes with a low AU-content site (bottom quartile). *D*, Efficacy of additional 3′ pairing. Plotted are the cumulative distribution of miRNA-mRNA and miRNA-ratio correlation between miRNAs and genes containing one 8mer site with good 3′ pairing (red) and that between miRNAs and genes with poor 3′ pairing (green) (*p* = 0.00209 for miRNA-mRNA, *p* = 0.00036 for miRNA-ratio, one-sided K-S test).

Messages down-regulated after introducing a miRNA are generally associated with four types of sites: 6mer, 7mer-m8, 7mer-A1, and 8mer (10). For genes with at least one 8mer site in their 3′UTRs, their mRNA abundance was significantly more likely to be negatively correlated with the expression of their cognate miRNAs, compared with genes containing no matched site (*p* < 1.0e-05, one sided KS-test, Fig. 3*A*). For genes with at least one 7mer-m8, 7mer-A1, or 6mer site in their 3′ UTRs, their mRNA abundance had a less pronounced, but still significant propensity to be more negatively correlated with the expression of cognate miRNAs compared with genes containing no site (*p* < 0.05, one-sided KS-test, Fig. 3*A*). These results mirrored findings from previous studies with selected miRNAs (10, 35) and confirmed that the 8mer site is most effective for driving mRNA decay.

However, this conclusion was not applicable to site efficacy in translational repression. Specifically, for genes with one or more 8mer sites in their 3′UTRs, their protein-to-mRNA ratios were not more negatively correlated with the expression of cognate miRNAs compared with genes containing no site (*p* > 0.5, one-sided KS-test, Fig. 3*A*). Thus, the 8mer site type was effective in boosting mRNA decay, but not translational repression.

##### Site Positioning Within the Gene Structure

Although the majority of investigations into miRNA function have been on sites located in 3′UTRs, 5′UTRs and open reading frames (ORFs) of mammalian genes also contain miRNA target sites. For genes with at least one 8mer site in their 3′UTRs, their mRNA abundance was more negatively correlated with the expression of cognate miRNAs compared with genes with no site (*p* < 1.0e-05, one sided KS-test, Fig. 3*B*). However, such correlation difference was marginally significant (*p* = 0.006, one sided KS-test) or not significant (*p* > 0.5, one sided KS-test) between genes with at least one 8mer site in their ORFs and 5′UTRs and genes with no site, respectively (Fig. 3*B*). These results agreed with previous miRNA transfection analysis, site-conservation analysis, and site depletion analysis (4, 10, 30, 36). For miRNA-ratio correlation, however, marginal significance was only observed for genes with at least one 8mer site in their ORFs (*p* = 0.012, one sided KS-test, Fig. 3*B*). This result further confirmed that 8mer sites located in 3′UTRs tend to drive mRNA decay, but not translational repression.

##### Local AU Rich Context

Previous studies have found that the nucleotides immediately flanking the functional sites are highly enriched for AU-content relative to nonfunctional sites (10). We used the scheme described by Grimson *et al.* (10) for scoring AU-content. The analysis was limited to genes with only one 8mer site. We ranked sites based on the local AU-content score from the highest to the lowest and defined the first quartile as high AU-content sites and the last quartile as low AU-content sites. For genes with a high AU-content 8mer site, their mRNA abundance was more negatively correlated with the expression of cognate miRNAs compared with genes with a low AU-content 8mer site (*p* = 0.047, one sided KS-test, Fig. 3*C*). A similar result was observed for miRNA-ratio correlation (*p* = 0.016, one sided KS-test, Fig. 3*C*). Therefore, AU-rich content enhanced both mRNA decay and translational repression, which should produce a stronger effect on protein level. As expected, genes with high AU-content sites showed a significantly more negative miRNA-protein correlation than genes with low AU-content sites (*p* = 0.0003, one sided KS-test), and the difference was more significant than those observed for miRNA-mRNA correlation and miRNA-ratio correlation (Fig. 3*C*).

##### Additional 3′ Pairing

Pairing to the 3′ portion of the miRNA is thought to enhance the efficacy of miRNA functional sites. We determined the 3′ pairing score as described by Grimson *et al.* (10). For miRNA-mRNA correlation, genes containing only one 8mer site with good 3′ pairing (3′ pairing score >4) were more negatively correlated with their cognate miRNAs than those with poor 3′ pairing (3′ pairing score <1) (*p* = 0.00209, one sided KS-test, Fig. 3*D*). Interestingly, an opposite trend was detected for miRNA-ratio correlation (*p* = 0.00036, one sided KS-test, Fig. 3*D*), indicating that additional 3′ pairing enhanced mRNA decay, but reduced translational repression. For a given miRNA and a given type of target site (8mer, 7mer-m8, 7mer-A1, and 6mer), miRNA-target relationships inducing mRNA decay (miRNA-mRNA correlation<-0.8) showed a significantly higher 3′ pairing score than those promoting translational repression (miRNA-ratio correlation<-0.8) (*p* = 0.004, paired *t* test for 144 miRNA and target site type combinations with paired data, supplemental Fig. S7), suggesting that reduced 3′ pairing may shift mRNA decay to translational repression.

##### miRNA-target Interactions in CRC Cell Lines

**Fig. 4.**
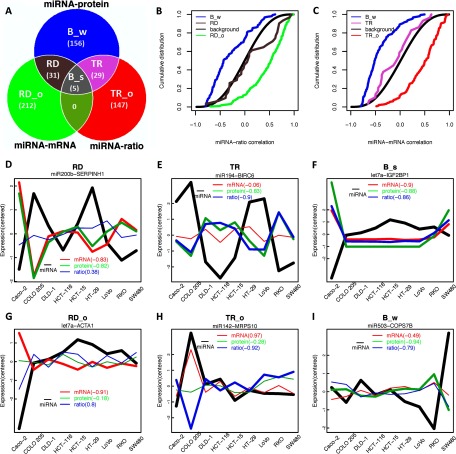
**Categorization of miRNA-target interactions.** *A*, Defining miRNA-target interaction categories based on the significance level of miRNA-mRNA, miRNA-protein and miRNA-ratio correlation. RD: m*R*NA *D*ecay; RD_o: m*R*NA *D*ecay with *o*ther mechanisms; TR: *T*ranslational *R*epression; TR_o: *T*ranslational *R*epression with *o*ther mechanisms; B_s: *B*oth *s*trong; B_w: *B*oth *w*eak. *B*, Cumulative distributions of miRNA-ratio correlation in categories RD, RD_o, B_w, and background (all miRNA-gene pairs), respectively. *C*, Cumulative distributions of miRNA-mRNA correlation in categories TR, TR_o, B_w, and background, respectively. *D–I*, Typical correlation patterns of miRNA-mRNA, miRNA-protein and miRNA-ratio in each category, RD (*D*), TR (*E*), B_s (*F*), RD_o (*G*), TR_o (*H*), and B_w (*I*). Plotted are the expression variation curves across nine cell lines for miRNA (black), mRNA (red), protein (green) and protein-to-mRNA ratio (blue). Bold lines suggest expressions significantly correlated with miRNA abundance. Three kinds of correlation coefficients were given in parenthesis.

To identify miRNA-target interactions, we combined functional evidence from the three types of significant inverse correlations (miRNA-mRNA, miRNA-protein, or miRNA-ratio) and binding evidence from sequence-based prediction tools including TargetScan (29, 30), miRanda (31), or MirTarget2 (32). Predictions made by TargetScan, miRanda, and MirTarget2 were retrieved from the RmiR.Hs.miRNA package (version 1.0.6). In total, we predicted 580 interactions, involving 60 miRNAs and 423 genes (supplemental Table S1 and supplemental Data Set S4). We further classified these interactions into six categories based on the type of supporting correlation (Fig. 4*A*), allowing us to infer the relative contributions of mRNA decay and translational repression to the interactions in each category.

1. RD (m*R*NA *D*ecay) (31 interactions). Significant inverse correlation was observed for miRNA-mRNA and miRNA-protein, but not for miRNA-ratio, suggesting protein changes closely reflected mRNA changes induced by miRNAs without additional translation efficiency changes (Fig. 4*D*). Thus, mRNA decay played the predominant role in these interactions.
2. RD_o (m*R*NA *D*ecay with *o*ther mechanisms) (212 interactions). Significant inverse correlation was observed for miRNA-mRNA, but not for miRNA-protein or miRNA-ratio, suggesting potential involvement of other regulatory mechanisms that produced a positive effect on translation to compensate for the miRNA-mediated mRNA decay (Fig. 4*G*). This proposition was supported by a positive shift in the cumulative distribution of miRNA-ratio correlation for these miRNA-target interactions as compared with the background distribution (miRNA-ratio correlation for all miRNA-gene pairs) (*p* < 1.0e-16, one sided KS-test, Fig. 4*B*). In contrast, the distribution of miRNA-ratio correlation for interactions in the RD category was almost the same as the background distribution (Fig. 4*B*). Thus, mRNA decay was the primary determinant for the interactions in the RD_o category, but other non-miRNA mediated mechanisms appeared to provide a compensatory positive effect on the translation of the target genes. (Fig. 4*G*).
3. TR (*T*ranslational *R*epression) (29 interactions). Significant inverse correlation was observed for miRNA-protein and miRNA-ratio but not for miRNA-mRNA, suggesting protein expression variation merely reflected miRNA directed translational efficiency changes without additional repression on mRNA abundance (Fig. 4*E*). Thus, translational repression played the predominant role in these interactions.
4. TR_o (*T*ranslational *R*epression with *o*ther mechanisms) (147 interactions). Significant inverse correlation was observed for miRNA-ratio, but not for miRNA-protein or miRNA-mRNA, suggesting that other transcriptional regulatory mechanisms provided a compensatory positive effect for miRNA-mediated translational repression (Fig. 4*H*). This proposition was supported by a positive shift in the cumulative distribution of miRNA-mRNA correlation for these miRNA-target interactions as compared with the background distribution (miRNA-mRNA correlation for all miRNA-gene pairs) (*p* < 1.0e-16, one sided KS-test, Fig. 4*C*). In contrast, miRNA-mRNA correlation for interactions in the TR category had almost the same distribution as the background (Fig. 4*C*). Thus, translational repression was the primary determinant in the TR_o category, but other transcriptional mechanisms nevertheless compensated the translational inhibition mediated by miRNA.
5. B_s (*B*oth *s*trong) (5 interactions). Both mRNA decay and translational repression contributed strongly to gene repression, leading to significant inverse correlations for miRNA-mRNA, miRNA-protein and miRNA-ratio (Fig. 4*F*).
6. B_w (*B*oth *w*eak) (156 interactions). Significant inverse correlation was observed for miRNA-protein, but not for miRNA-mRNA or miRNA-ratio, suggesting that both mRNA decay and translational repression had a weak contribution to gene repression, but their combined effect led to a strong impact on protein product (Fig. 4*I*). This proposition was supported by a negative shift of the cumulative distribution curves of miRNA-mRNA and miRNA-ratio correlations for these miRNA-target interactions as compared with the background distributions (Figs. 4*B* and 4*C*, *p* < 1.0e-16, one sided KS-test), indicating weak repression effect by miRNA through both mRNA decay and translational repression.

There was no miRNA-target interaction belonging to the category where significant inverse correlation was observed for miRNA-mRNA and miRNA-ratio, but not miRNA-protein because significant inverse miRNA-protein correlation should be detected given significant inverse miRNA-mRNA and miRNA-ratio correlations (Fig. 4*A*).

Although 248 out of the 580 interactions (43%) could be identified based solely on transcriptomics data (RD, RD_o, and B_s categories), 332 interactions (57%) benefited from the integration of proteomics data (B_w, TR, and TR_o categories). Thus, the proteomics data were absolutely required for a comprehensive assessment of miRNA mediated regulation. Translational repression played a major role in 30% of the interactions (176/580, TR and TR_o categories). Another 28% of the interactions (161/580, B_w and B_s categories) involved concordant mRNA decay and translational repression (Fig. 4*A*). These results revealed that translational repression played an equally important role as mRNA decay in miRNA-mediated regulation, which was in contrast to recent large studies suggesting a dominant role of mRNA decay (2, 3, 8, 9). Because 7–8mer sites were found to favor mRNA decay rather than translational repression, the relative contribution of mRNA decay might be overestimated in recent studies which reached the conclusion based primarily on the response of genes with canonical 7–8 mer sites in 3′UTRs.

**Fig. 5.**
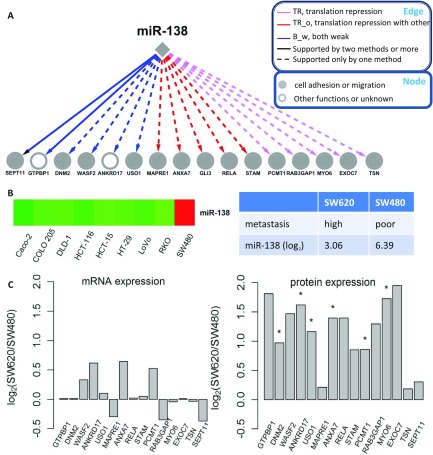
**miR-138 and its target genes.** *A*, Interactions between miR-138 and its target genes. Edges and nodes are annotated in the boxes. Edge color represents interaction category defined in this study; edge type represents level of supporting from sequence-based methods including TargetScan, miRanda, and MirTarget2; node color represents functional annotation. *B*, Expression data of miR-138 in different cell lines. Red and green represent relative high- and low-expression, respectively. *C***,** Relative expression of miR-138 and its target genes in SW620 *versus* SW480 as measured by log_2_ ratio (*FDR<0.05, Poisson regression model).

Furthermore, we found that translational repression was the predominant contributor to miR-138 mediated regulation (FDR<0.05, Fisher exact test). Among the 16 targets regulated by miR-138, ten were mainly inhibited by translational repression (TR or TR_o category) and the remaining six were repressed by both weak mRNA decay and translational repression (B_w category), whereas none of the targets were mainly silenced by mRNA decay (RD or RD_o category), suggesting a preference of miR-138 for triggering translational repression (Fig. 5*A* and supplemental Table S1). Among the nine cell lines, miR-138 was highly expressed in SW480, a nonmetastatic cell line (Fig. 5*B*). We downloaded miRNA and mRNA expression data for its genetically matched cell line, SW620 from GEO (GSE10833 and GSE10843). SW480 and SW620 were derived from primary tumors and distant metastases from the same patient, respectively. An eightfold overexpression of miR-138 was observed in SW480 compared with SW620; however, none of the targets showed a greater than 2-fold up-regulation in SW620 on mRNA expression (Fig. 5*C*). In contrast, in a follow-up shotgun proteomics experiment comparing SW480 and SW620 proteomes, all of the targets showed higher protein abundance in SW620 except for GLI3, which was not detected in SW620 by shotgun proteomics. Nine out of the 15 targets (GTPBP1, WASF2, ANKRD17, USO1, ANXA7, RELA, RAB3GAP1, MYO6, and EXOC7) were up-regulated by more than twofold in SW620 and 6 targets (DNM2, ANKRD17, USO1, ANXA7, PCMT1, and MYO6) showed statistically significant up-regulation (FDR < 0.05, Poisson regression model, Fig. 5*C*). These results further demonstrated that translational repression was the primary determinant in miR-138 mediated regulation. Consistent with this, a previous study found that the inhibitory effect of miR-138 on APT1 expression is due mainly to impaired translation of APT1 mRNA (37).

In addition to revealing the important role of translational repression at a global scale and in specific miRNA directed gene inhibition, our integrative omics analysis also helped identify biologically meaningful interactions and suggest potential roles and downstream effectors of miRNAs in human colon cancer. For example, among the 16 targets regulated by miR-138, 14 are involved in cell migration or tumor metastasis (Fig. 5*A*), suggesting a possible role of miR-138 in metastasis. This was consistent with its significant differential expression between the non-metastatic cell line SW480 and the metastatic cell line SW620. In addition, previous knockdown experiments in head and neck squamous cell carcinoma cell lines have demonstrated its role in suppressing cell invasion (38, 39).

**Fig. 6.**
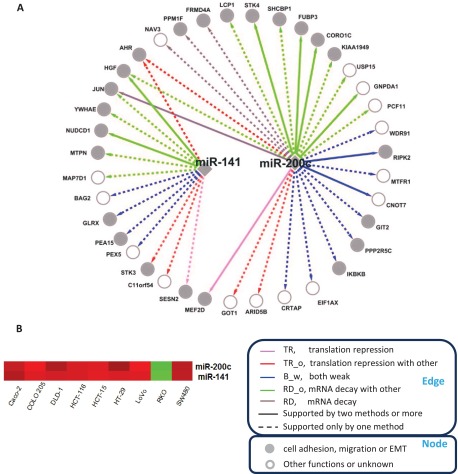
**miR-141, miR-200c and their target genes.** *A*, Interactions between miR-141, miR-200c and their target genes. Edges and nodes are annotated in the boxes. Edge color represents interaction category defined in this study; edge type represents level of supporting from sequence-based methods including TargetScan, miRanda and MirTarget2; node color represents functional annotation. *B*, Expression data for these two miRNAs in different cell lines. Red and green represent relative high- and low-expression, respectively.

As another example, ten out of the 14 targets regulated by miR-141 and 16 out of the 27 targets by miR-200c are involved in cell adhesion, migration or epithelial-mesenchymal transition (EMT) (Fig. 6*A*), which agrees with previously reported roles for these two miRNA-200 family members (40–43). Compared with other cell lines, these two miRNAs were down-regulated in the RKO cell line (Fig. 6*B*), which has gained a mesenchymal phenotype through EMT (44). Indeed, a strong inverse correlation between miRNA-200 family and the EMT transcriptional program was recently reported in a study based on a large human CRC cohort with 326 tumors (45).

## DISCUSSION

We have performed a comprehensive investigation of miRNA-mediated regulation through an integrative analysis of the endogenous variation of miRNA, mRNA and protein expression in multiple cell lines. Translational repression was involved in 58% (TR, TR_o, B_w, and B_s categories) and played a major role in 30% (TR and TR_o categories) of all predicted miRNA-target interactions. It is possible that translational repression may rapidly lead to mRNA decay and result in nonobservable effects on the protein-to-mRNA ratio. Therefore, we cannot rule out a possible contribution of translational repression to interactions in the RD and RD_o categories. Taken together, our results provide a clearer understanding of the importance and scope of translational repression in miRNA-mediated regulation in colon cancer cell lines. It would be of high interest to investigate whether these observations could be extended outside of cancer cell lines in future studies.

A somewhat unexpected result is that sequence features known to drive site efficacy in mRNA decay are generally not applicable to translational repression. We found that 8mer site type, site positioning within 3′ UTR, high local AU-rich context and good 3′ pairing all helped increase site efficacy in mRNA decay. This result echoes the finding by Grimson *et al.* (10), in which changes in mRNA abundance after miRNA transfection were used to evaluate site efficacy. To our knowledge, no work has described sequence features affecting site efficacy in translational repression. By dissecting the relative contribution of mRNA decay and translational repression, we found that among the above features, only high local AU-rich context increased translational repression. High local AU-rich context creates a more accessible UTR structure (35), which may benefit both mRNA decay and translational repression mechanisms. Although 8mer sites in 3′UTRs are most effective for promoting mRNA decay, they had little effect on boosting translational repression. Our data also showed that good 3′ pairing boosted site efficacy in mRNA decay, but reduced site efficiency in translational repression.

A key unanswered question regarding miRNAs is what determines selectivity for mRNA decay *versus* translational repression. Our data indicate that perfect matches to both the seed and 3′ portion of miRNA favor mRNA decay more than translational repression, whereas structural subtleties of imperfect miRNA-mRNA duplexes may preferentially trigger translational repression. In a small-scale study on small interfering RNAs (siRNAs) using reporter constructs, Aleman *et al.* found that a perfect match to positions 9–11 of the siRNA is critical for siRNA-mediated cleavage of mRNAs, but is not required for the repression of protein production (46). In a recent large-scale study, Selbach *et al.* also found that mismatches in this region are deleterious to miRNA-mediated mRNA decay, but they correlate with increased repression of protein production by miRNAs (3). Our results also suggest that, besides mRNA sequence, the preference for mRNA decay or translational repression may be largely dictated by the miRNA itself, as evidenced by strong translational repression preference of miR-138. One explanation is that a specific miRNA may preferentially assemble with a particular Argonaute protein (47), which may contribute to determine triggering a specific mechanism (48, 49).

The relative contribution of mRNA decay and translational repression to miRNA-mediated regulation in mammalian cells has been a topic of considerable debate. Early studies favored a translational repression-centric scenario (6, 7), whereas some recent studies suggest an mRNA decay-centric scenario (2, 8). In a most recent study, Guo *et al.* (8) used ribosome profiling to measure the overall effect of miRNAs on protein production and compared that with simultaneously measured effects on mRNA levels. They found that changes in mRNA levels closely reflect the impact of miRNAs on protein production and thus concluded that mRNA decay is the predominant driver of reduced protein output. It should be noted that this conclusion was based on genes with at least one canonical 7–8 mer site, for which intense regulation of mRNA decay has been previously reported and was confirmed in our study (Fig. 3*A*). In contrast, much weaker regulation of translational repression was observed for genes with either 8 mer sites or 7 mer sites (Fig. 3*A*). One explanation is that 7–8mer sites favor mRNA decay more than translational repression, which leads to a predominant role of mRNA decay for genes with at least one 7–8mer site. Consistently, we found that miRNA-gene pairs detected by miRNA-mRNA correlation were better supported by TargetScan than those detected by miRNA-ratio correlation, possibly because TargetScan requires an exact match to the seed sequence (29, 30). Previous studies also found that many proteins with altered protein expression after miRNA perturbation were not predicted as targets by existing algorithms (3, 50). Thus, known sequence features mainly mediate mRNA decay and our limited understanding of sequence features that mediate translational repression might have led to an underestimation of the contribution from translational repression. The miRNA-gene pairs with significant inverse miRNA-ratio correlation detected in our analysis provide good candidates for further computational analysis to identify translational repression-related sequence features. Moreover, miR-138 may serve as a good experimental model for better understanding miRNA-mediated translational repression, because of its strong preference for this mechanism. Our correlation-based analysis provides an intuitive, but powerful means that allows efficient data integration for delineating systems-wide behavior and generating specific hypotheses that can be further confirmed by more focused miRNA perturbation studies.

## Supplementary Material

Supplemental Data

## Footnotes

^1^ The abbreviations used are:

CRC: colorectal cancer
FDR: false discovery rate
TR: translational repression
TR_o: TR with other mechanisms
B_w: both weak
B_s: both strong
RD: mRNA decay.
