# Supplementary material for: Integrative Omics Analysis Reveals the Importance and Scope of Translational Repression in microRNA-mediated Regulation
Source: Mol Cell Proteomics. 2013 Apr 2;12(7):1900–11. doi: 10.1074/mcp.M112.025783 (PMC3708174; doi:10.1074/mcp.M112.025783)
Supplement: Supplemental Data [file supp_12_7_1900__index.html]

Integrative omics analysis reveals the importance and scope of translational repression in microRNA-mediated regulation — Integrative Omics Analysis Reveals the Importance and Scope of Translational Repression in microRNA-mediated Regulation — Importance and Scope of Translational Repression in microRNA-mediated Regulation — Supplemental Data 

# Integrative Omics Analysis Reveals the Importance and Scope of Translational Repression in microRNA-mediated Regulation

## Supplemental Data

**Files in this Data Supplement:**

- Supplementary file 1 - This file contains figures S1-S7, text S1 and table S1.
- Supplemental Data Set S1 - Peptide and protein identification.
- Supplemental Data Set S2 - Protein quantification data.
- Supplemental Data Set S3 - Sequence features in miRNA-gene pairs.
- Supplemental Data Set S4 - miRNA-target interactions.
