# Supplementary material for: Integrative Omics Analysis Reveals the Importance and Scope of Translational Repression in microRNA-mediated Regulation
Source: Mol Cell Proteomics. 2013 Apr 2;12(7):1900–11. doi: 10.1074/mcp.M112.025783 (PMC3708174; doi:10.1074/mcp.M112.025783)
Supplement: Supplemental Data [file supp_M112.025783_mcp.M112.025783-1.pdf]

Integrative omics analysis reveals the importance and scope of translational repression in microRNA-mediated regulation

Qi Liu[*], Patrick J Halvey[*], Yu Shyr, Robbert J C Slebos, Daniel C Liebler[§], Bing Zhang[§]

Supplementary materials

Supplementary File 1

This file contains figures S1-S7, text S1 and table S1.

Supplemental Data Set S1 – Peptide and protein identification

This xml file generated by IDPicker and relevant tables derived from the xml file. These files include detailed information on peptide and protein identification, protein inference from peptide assignments, and quantification.

Supplemental Data Set S2 – Protein quantification data

This spreadsheet lists the total spectral count normalized and log transformed protein abundance for the 5144 genes in the 9 cell lines.

Supplemental Data Set S3 – Sequence features in miRNA-gene pairs

This spreadsheet lists the 3' UTR length of the gene, number of target sites, type of target sites, local AU-context score, and additional 3' pairing score for all 406376 (79*5144) miRNA-gene pairs.

Supplemental Data Set S4 – miRNA-target interactions

This spreadsheet describes 580 miRNA-target interactions with the categorization and supporting information from TargetScan, miRanda and MirTarget2.

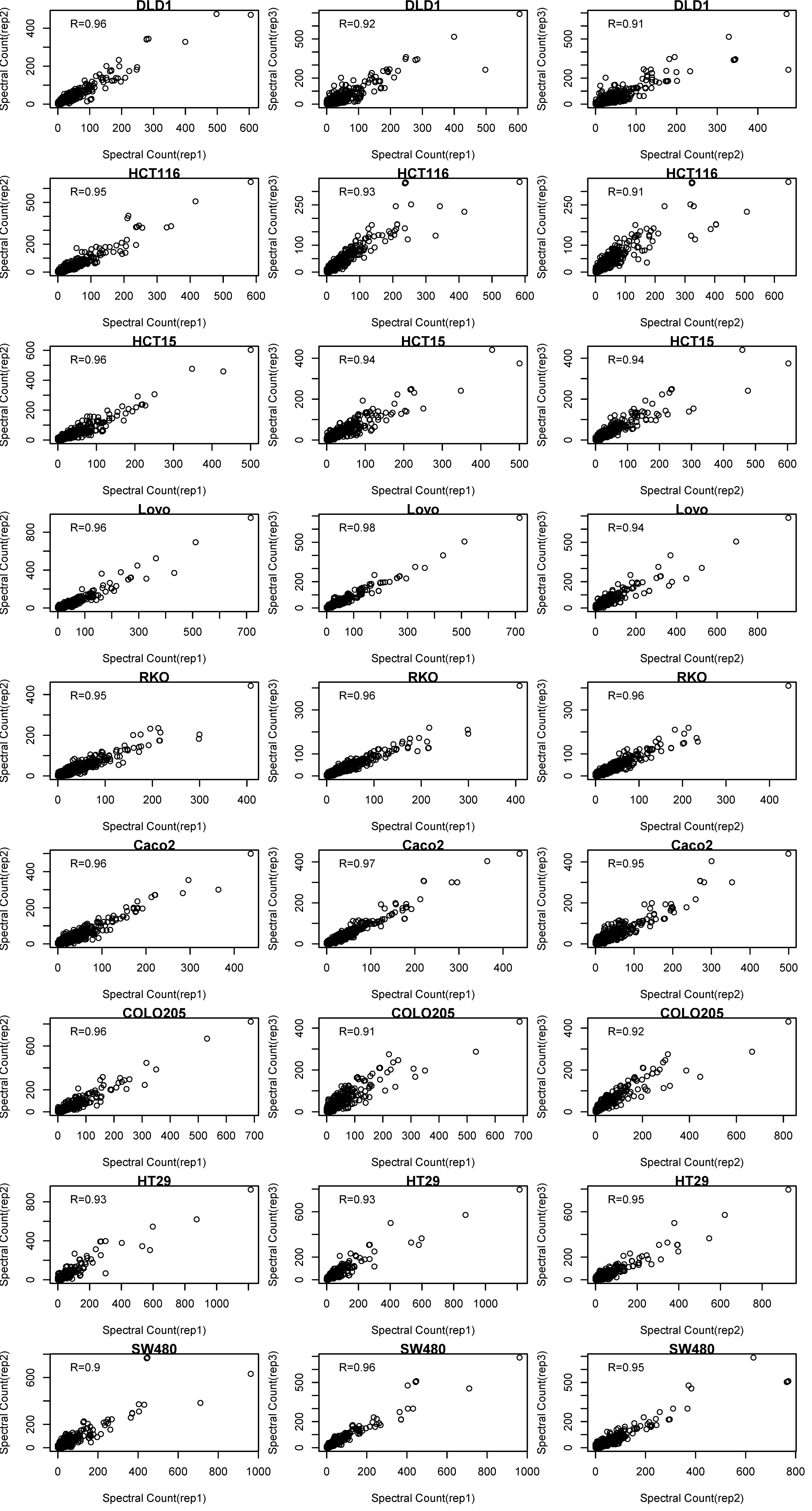

Fig. S1. Reproducibility of protein concentration measurements. Spectral counting based methods of protein concentration measurements are highly reproducible. Spectral counts of replicate measurements in each cell line are highly correlated (R=0.9~0.98 with p-value<2e-16, N=6124).

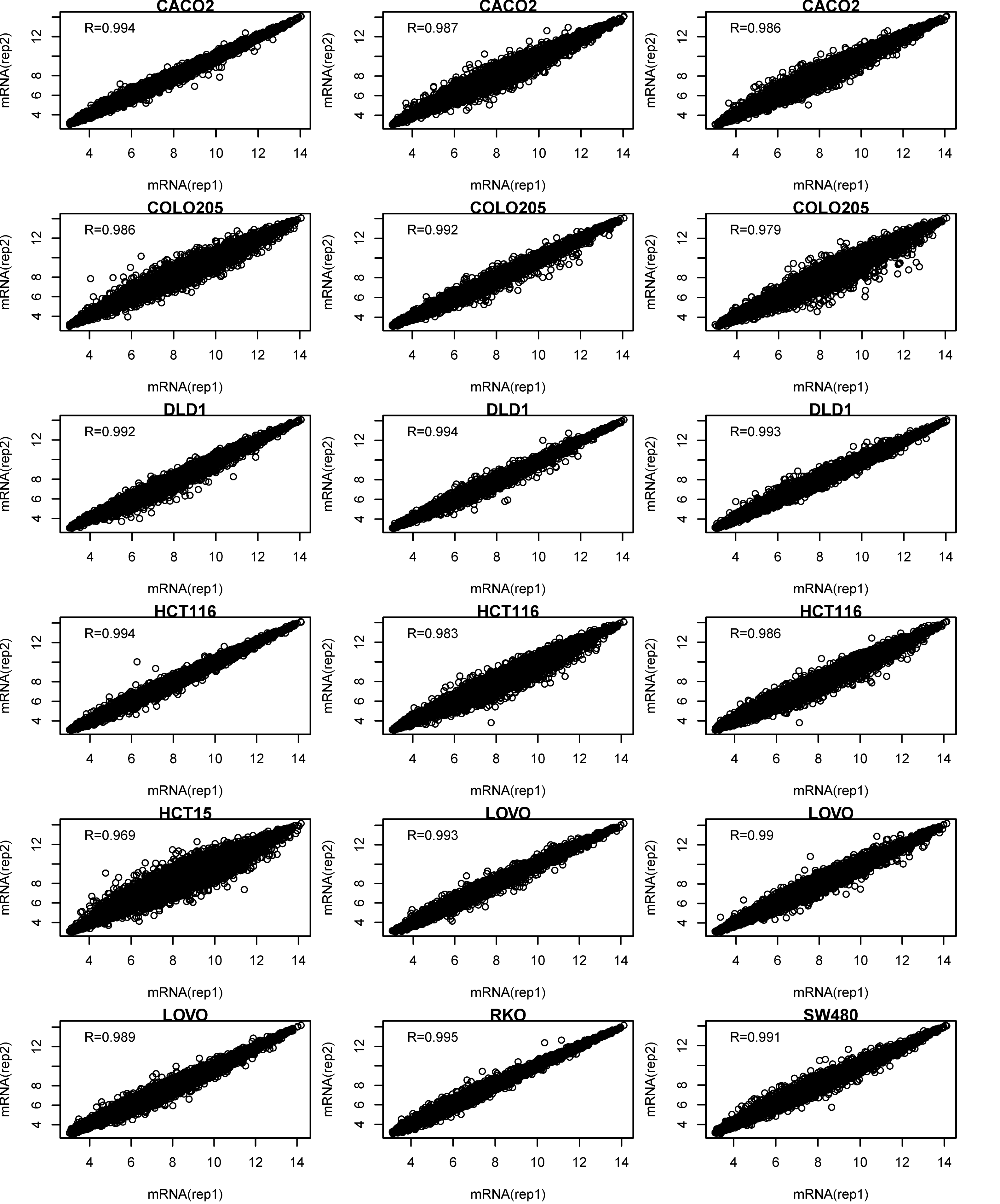

Fig. S2. Reproducibility of mRNA concentration measurements. There were 3 replicates for Caco-2, COLO 205, DLD-1, LoVo and HCT-116 cell lines, 2 replicates for HCT-15, RKO and SW480 cell lines, and only 1 replicate for HT-29 cell line. The correlation between (log_2) intensities of replicate measurements in each cell line lies between 0.96 and 0.99 (p-value<2e-16, N= 19468).

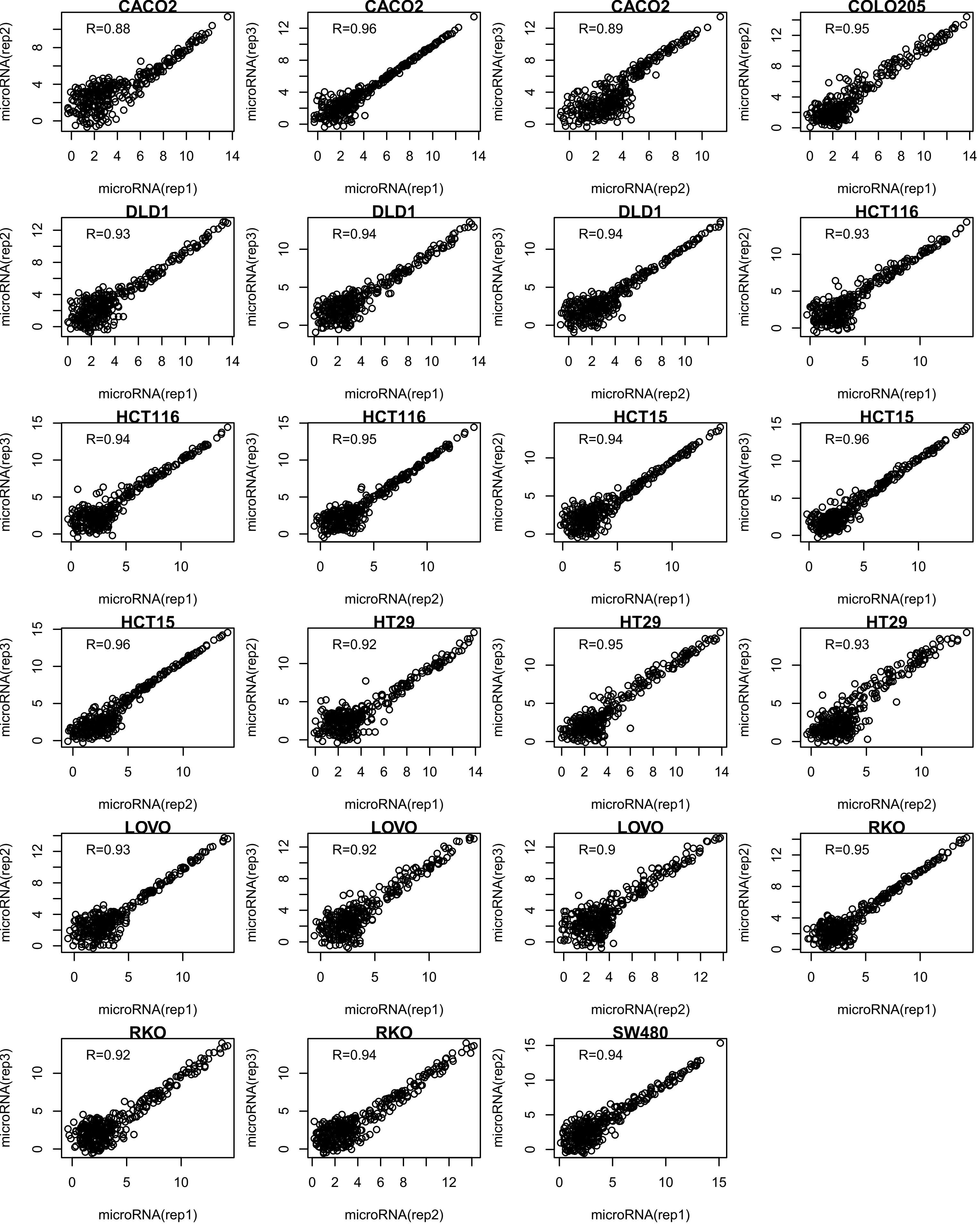

Fig. S3. Reproducibility of microRNA concentration measurements. There were 2 replicates for COLO 205 and SW480 cell lines, and 3 replicates for HT-29, HCT-15, Caco-2, DLD-1, RKO, LoVo and HCT-116 cell lines. The correlation between (log_2) intensities of replicate measurements in each cell line lies between 0.88 and 0.95 (p-value<2e-16, N=328).

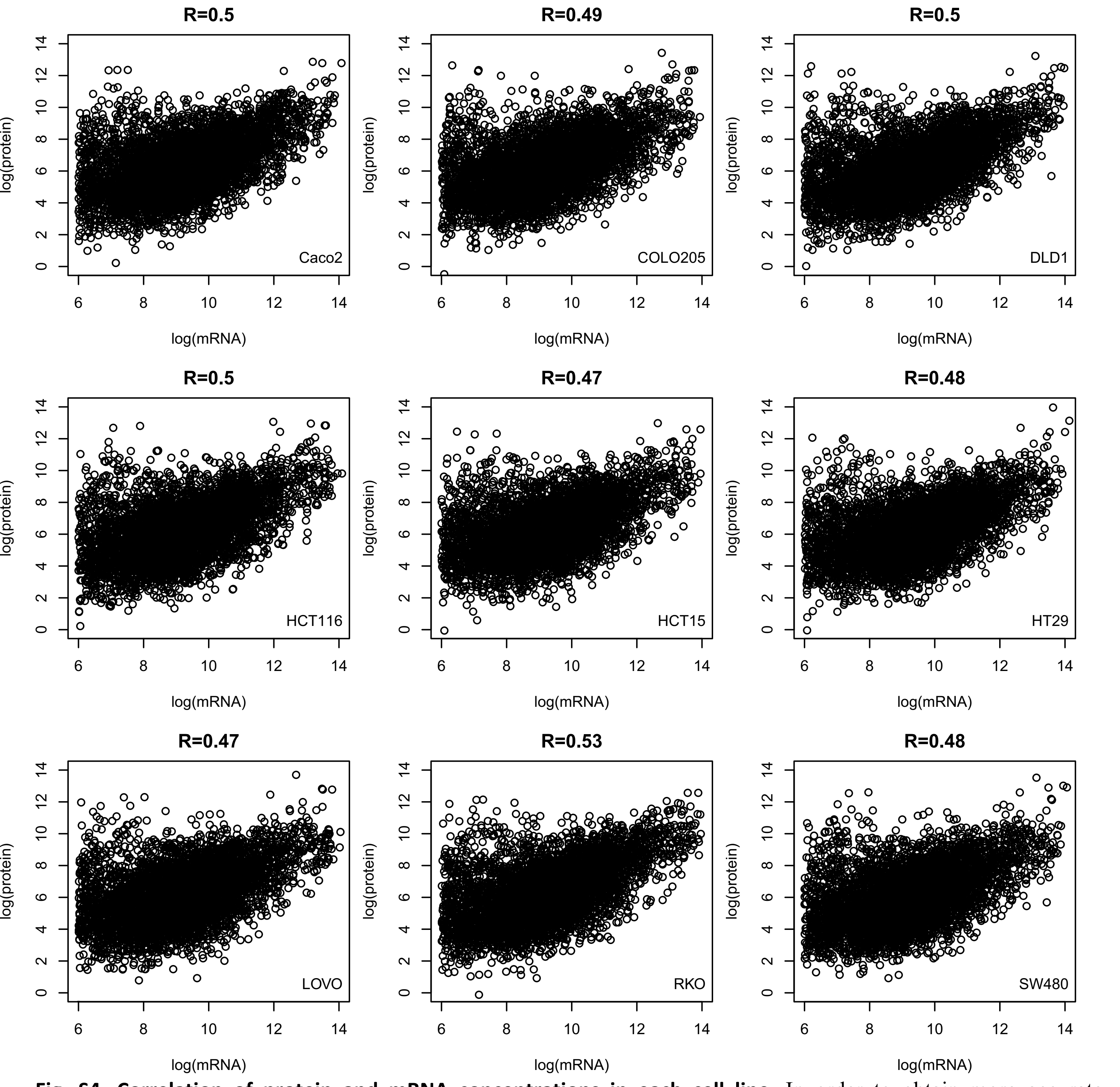

Fig. S4. Correlation of protein and mRNA concentrations in each cell line. In order to obtain more accurate estimation of the correlation between protein and mRNA concentration, protein abundance was further normalized for the protein length to 10000aa. Protein and mRNA concentrations correlate significantly at a log_2-log_2 scale. We removed outliers with log_2 (mRNA)<6 according to the mRNA concentration distribution. (N=4708~4829, R=0.47~0.53 with p-value<2e-16).

Text S1

The usefulness of three types of correlation

To assess whether three types of correlation between miRNAs and genes (miRNA-mRNA, miRNA-protein and miRNA-ratio) could help find true miRNA targets, we evaluated the functional coherence of genes significantly correlated with the same miRNA as well as evolutionary conservation of the 6mer seed match regions. First, we compared the pairwise functional similarity for genes significantly inverse correlated with the same miRNA with that for random genes. Three types of functional similarity measurements were calculated based on different categories of Gene Ontology (GO) annotations: molecular function, biological process, and cellular component. Figure S5 shows pairwise functional similarity for genes significantly inverse correlated to a common miRNA at the mRNA expression level (miRNA-mRNA correlation, red), the protein expression level (miRNA-protein correlation, blue), the protein-to-mRNA ratio level (miRNA-ratio correlation, green) was significantly higher than that for random genes ($p<0.005$ for all comparisons, one sided KS-test, Fig. S5). Moreover, miRNA-protein correlation led to the most significant increase in pairwise functional similarity ($p<1.0e-15$ for all types of similarity measurements, one sided KS-test, Fig. S5). These results suggested that all three types of significant inverse correlation helped enrich biologically meaningful miRNA-gene relationships.

On the other hand, correlation based inference identify both direct and indirect targets of miRNAs. The miRNA seed region, i.e., miRNA positions 2-7, is the primary determinant of miRNA target recognition, and thus, a prefect 6nt match to the miRNA seed in the 3’ UTR of transcripts can be used as a preliminary screen to search for direct miRNA targets. We found that in general, the 6mer seed match regions were more conserved than their neighboring regions (i.e.,

50nt upstream and 50nt downstream, Fig. S6). Furthermore, compared with regions from genes without significant correlation to corresponding miRNAs, those from genes with any kinds of significant inverse correlation to corresponding miRNAs were significantly more conserved ($p<1.0e-05$, $p=0.05$, and $p=0.003$ for the miRNA-mRNA, miRNA-protein, and miRNA-ratio, respectively, one sided KS-test). Thus all three kinds of significant inverse correlation helped enrich genes containing conserved 6mer seed match sites, i.e., genes more likely to be miRNA direct targets.

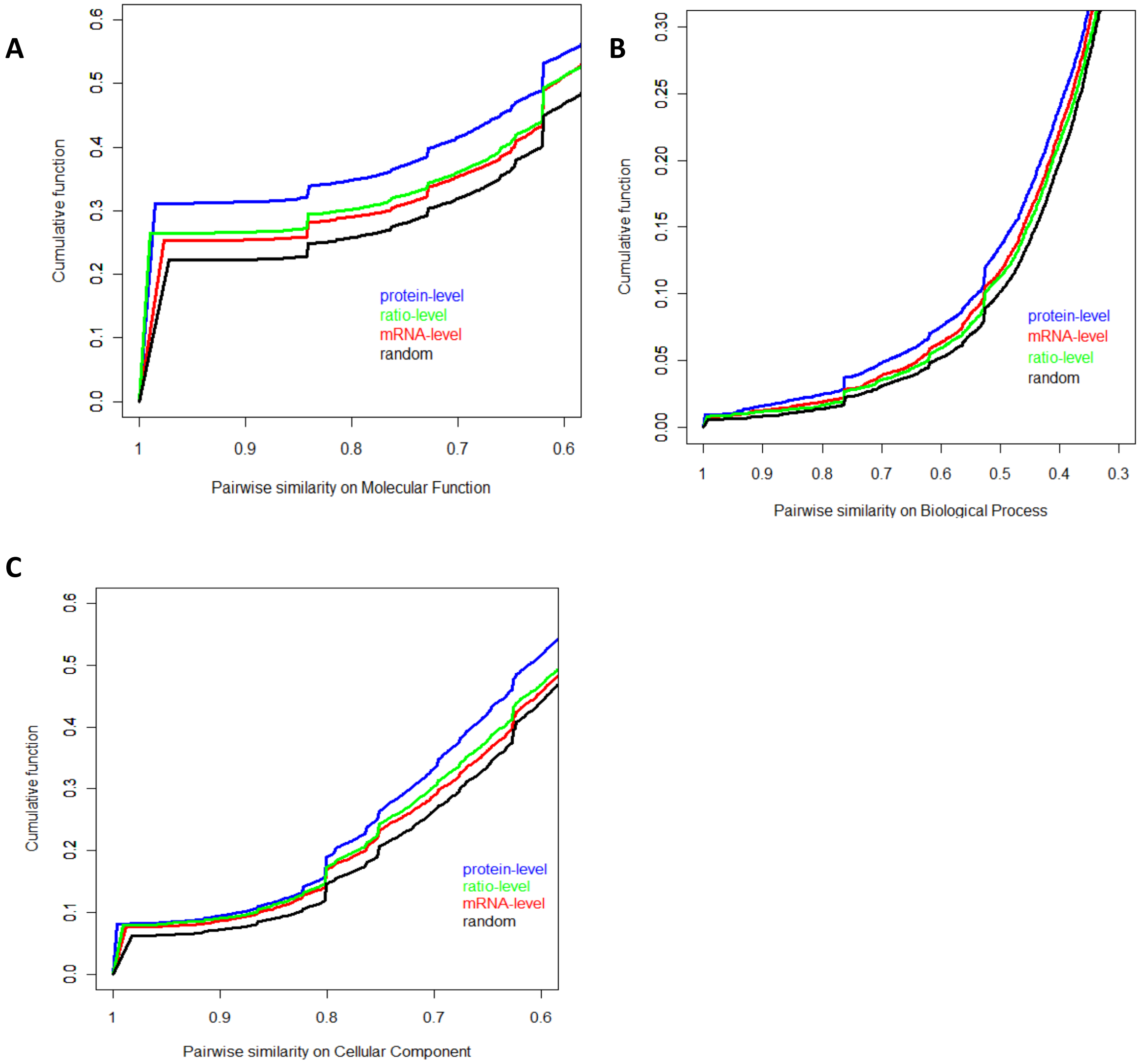

Fig. S5. Increased functional coherence was observed for genes showing significantly inverse correlation at the mRNA-level (miRNA-mRNA correlation, red), the protein-level (miRNA-protein correlation, green), and the ratio-level (miRNA-ratio correlation, blue). Functional similarity was measured based on three types of Gene Ontology annotations: **(A)** Molecular function. **(B)** Biological process. **(C)** Cellular component. Plotted are the cumulative distribution of pairwise functional similarity for genes correlated with a common miRNA at the mRNA expression level (red), protein expression level (blue), and the protein-to-mRNA ratio level (green), and that for random gene pairs (black). Genes correlated with a common miRNA at the mRNA level, the protein level or the ratio level are significantly functional coherent than random gene pairs (p<0.005, p<1.0e-15, p<0.005, respectively).

A

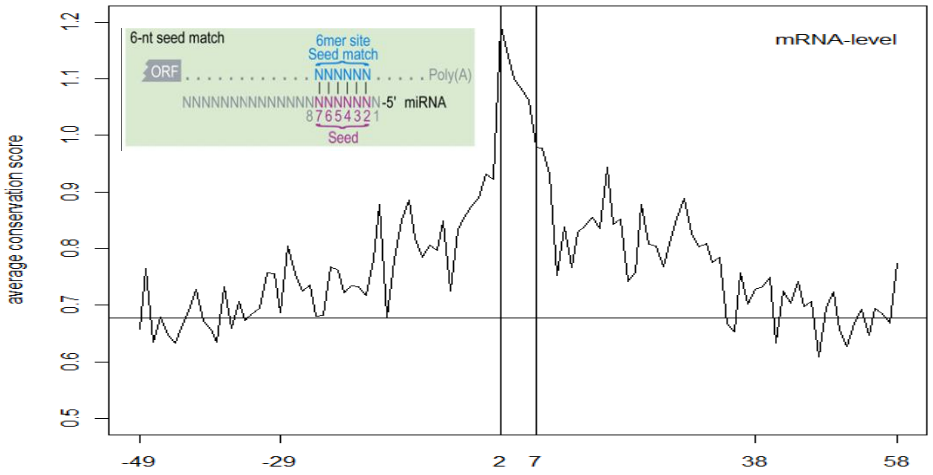

B

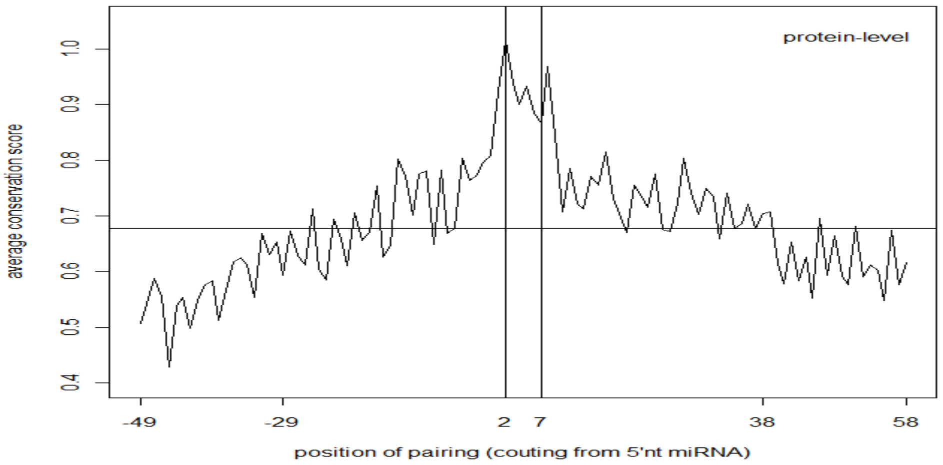

C

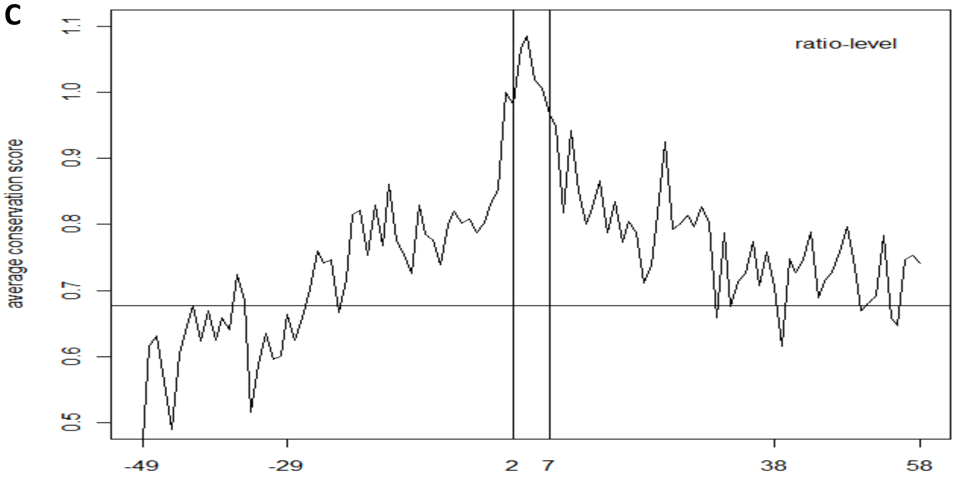

Fig. S6. 6 mer seed matched sites are more conserved than their neighboring regions. (A) 6mer seed matched sites obtained from genes with significant inverse correlation to cognate miRNAs at mRNA expression level (miRNA-mRNA correlation). **(B)** 6mer seed matched sites obtained from genes with significant inverse correlation to cognate miRNAs at protein expression level (miRNA-protein correlation). **(C)** 6mer seed matched sites obtained from genes with significant inverse correlation to cognate miRNAs at protein-to-mRNA ratio level (miRNA-ratio correlation).

Functional coherence and conservation analysis

Functional similarity between genes were calculated using the GOSemSim-package (1). The method proposed by Wang et al. (2) was selected to measure the semantic similarity of Gene Ontology terms.

Basewise conservation scores (phyloP) based on multiple alignments of 45 vertebrate genomes with human were downloaded from http://hgdownload.cse.ucsc.edu/goldenPath/hg19/phyloP46way/vertebrate/ and converted to the bigWig format using wigToBigWig. R scripts were generated to retrieve regions of interest from

the bigWig version using bigWigSummary. wigToBigWig and bigWigSummary were downloaded from http://hgdownload.cse.ucsc.edu/admin/exe/. Human 3'UTRs were scanned for sites with at least a 6mer seed match to their cognate miRNAs. The conservation scores for the 6mer seed match regions, the upstream 50 nt and the downstream 50 nt regions were retrieved. When a 3'UTR had more than one 6mer seed matches to a cognate miRNA, the 6mer seed match region with the highest average conservation scores was used.

1. Yu, G., Li, F., Qin, Y., Bo, X., Wu, Y., and Wang, S. (2010) GOSemSim: an R package for measuring semantic similarity among GO terms and gene products. *Bioinformatics* 26, 976-978
2. Wang, J. Z., Du, Z., Payattakool, R., Yu, P. S., and Chen, C. F. (2007) A new method to measure the semantic similarity of GO terms. *Bioinformatics* 23, 1274-1281

Table S1. Summary of miRNA-target interactions in CRC cell lines.

miRNA	Num of Targets	Num of targets in each category					
		TR_o	B_w	TR	RD_o	RD	B_s
hsa-let-7a	20	5	7	0	5	2	1
hsa-let-7b	25	6	10	1	5	2	1
hsa-let-7c	26	5	10	1	6	3	1
hsa-let-7d	21	5	7	0	6	2	1
hsa-let-7e	1	0	1	0	0	0	0
hsa-let-7f	17	4	4	0	5	4	0
hsa-let-7i	15	3	1	0	11	0	0
hsa-miR-100	2	1	0	0	1	0	0
hsa-miR-10a	5	2	3	0	0	0	0
hsa-miR-10b	2	1	1	0	0	0	0
hsa-miR-125b	4	2	1	0	0	1	0
hsa-miR-126	2	0	1	0	1	0	0
hsa-miR-128a	2	1	1	0	0	0	0
hsa-miR-130a	5	1	2	0	2	0	0
hsa-miR-132	10	2	1	0	7	0	0
hsa-miR-136	14	4	0	0	10	0	0
hsa-miR-138	16	5	6	5*	0	0	0
hsa-miR-141	14	3	4	1	6	0	0
hsa-miR-142-5p	9	1	5	0	3	0	0
hsa-miR-145	6	4	2	0	0	0	0
hsa-miR-146a	14	3	1	0	10	0	0
hsa-miR-148a	3	0	1	0	2	0	0
hsa-miR-152	3	1	1	0	1	0	0
hsa-miR-155	2	1	0	0	1	0	0
hsa-miR-181b	13	5	1	3	4	0	0
hsa-miR-18a	4	3	1	0	0	0	0
hsa-miR-18b	5	4	1	0	0	0	0
hsa-miR-192	6	0	1	0	5	0	0
hsa-miR-193b	1	1	0	0	0	0	0
hsa-miR-194	16	1	3	1	11	0	0
hsa-miR-195	11	3	3	0	4	1	0
hsa-miR-196a	4	0	1	1	2	0	0
hsa-miR-196b	9	2	3	0	4	0	0
hsa-miR-197	6	4	0	1	1	0	0
hsa-miR-200a	11	1	5	0	4	1	0
hsa-miR-200b	15	3	3	2	4	3	0
hsa-miR-200c	27	3	9	1	10	4	0

hsa-miR-202	6	1	2	0	3	0	0
hsa-miR-203	4	2	1	0	1	0	0
hsa-miR-205	14	3	3	2	6	0	0
hsa-miR-215	4	0	1	0	3	0	0
hsa-miR-221	10	6	3	0	1	0	0
hsa-miR-222	7	2	2	0	3	0	0
hsa-miR-224	13	5	3	3	2	0	0
hsa-miR-27b	8	1	0	0	7	0	0
hsa-miR-29a	17	3	4	1	7	2	0
hsa-miR-30b	12	3	1	1	7	0	0
hsa-miR-31	5	1	0	0	4	0	0
hsa-miR-34a	4	0	0	0	4	0	0
hsa-miR-372	7	1	1	0	5	0	0
hsa-miR-373	11	1	1	0	8	1	0
hsa-miR-375	1	1	0	0	0	0	0
hsa-miR-429	17	8	6	2	1	0	0
hsa-miR-452	22	4	8	1	9	0	0
hsa-miR-503	15	5	8	1	1	0	0
hsa-miR-505	2	1	0	0	1	0	0
hsa-miR-7	9	2	3	1	2	1	0
hsa-miR-95	4	1	3	0	0	0	0
hsa-miR-98	21	5	5	0	6	4	1
hsa-miR-99a	1	1	0	0	0	0	0
total	580	147	156	29	212	31	5

RD: mRNA-Decay, significant negative correlation detected both at the mRNA-level and the protein-level; RD_o: mRNA-Decay with other mechanisms, significant negative correlation detected only at the mRNA-level; TR: Translational Repression, significant negative correlation detected both at the ratio-level and the protein-level; TR_o: Translational Repression with other mechanisms, significant negative correlation detected only at the ratio-level; B_s: Both strong, significant negative correlation detected at three levels; B_w: Both weak, significant negative correlation detected only at the protein-level; *FDR<0.05.

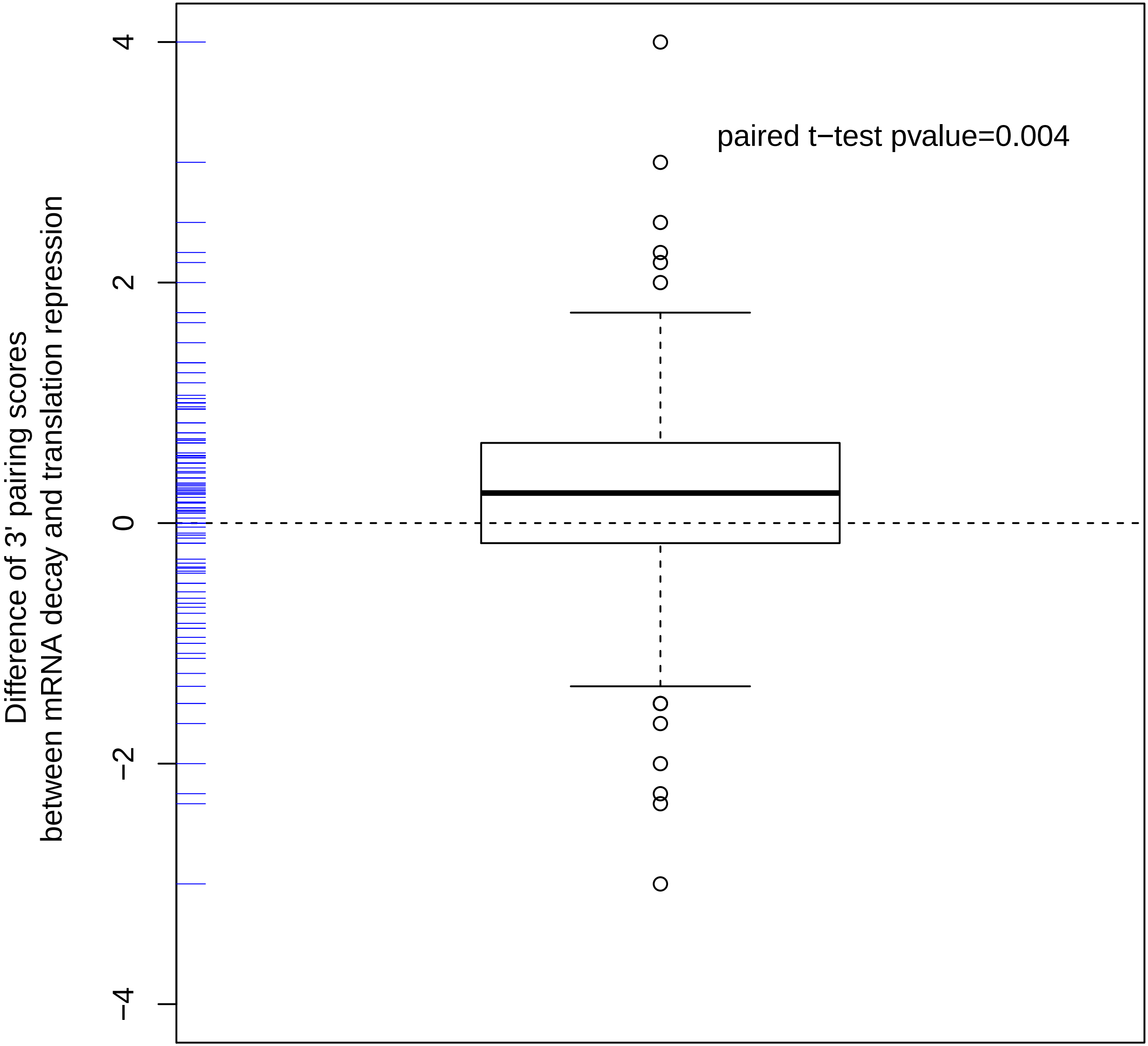

Fig. S7. Difference of 3' pairing scores between miRNA-target interactions driving mRNA decay and those promoting translational repression.
